# Circulating CXCR5^−^PD-1^hi^ peripheral T helper cells are associated with progression to type 1 diabetes

**DOI:** 10.1007/s00125-019-4936-8

**Published:** 2019-07-03

**Authors:** Ilse Ekman, Emmi-Leena Ihantola, Tyyne Viisanen, Deepak A. Rao, Kirsti Näntö-Salonen, Mikael Knip, Riitta Veijola, Jorma Toppari, Jorma Ilonen, Tuure Kinnunen

**Affiliations:** 1 Department of Clinical Microbiology, Institute of Clinical Medicine, University of Eastern Finland, Yliopistonranta 1 C, FIN-70210 Kuopio, Finland; 2 Division of Rheumatology, Immunology, and Allergy, Brigham and Women’s Hospital and Harvard Medical School, Boston, MA USA; 3 Department of Pediatrics, Turku University Hospital, Turku, Finland; 4 Tampere Center for Child Health Research, Tampere University Hospital, Tampere, Finland; 5 Children’s Hospital, University of Helsinki and Helsinki University Hospital, Helsinki, Finland; 6 Research Programs Unit, Diabetes and Obesity, University of Helsinki, Helsinki, Finland; 7 Folkhälsan Research Center, Helsinki, Finland; 8 Department of Pediatrics, Medical Research Center, PEDEGO Research Unit, Oulu University Hospital and University of Oulu, Oulu, Finland; 9 Institute of Biomedicine, Research Centre for Integrative Physiology and Pharmacology, University of Turku, Turku, Finland; 10 Immunogenetics Laboratory, Institute of Biomedicine, University of Turku, Turku, Finland; 11 Clinical Microbiology, Turku University Hospital, Turku, Finland; 12 Eastern Finland Laboratory Centre (ISLAB), Kuopio, Finland

**Keywords:** Autoimmunity, B cells, Follicular T helper cell, Human, Immunophenotyping, Peripheral T helper cell, T cells, Type 1 diabetes

## Abstract

**Aims/hypothesis:**

Type 1 diabetes is preceded by a period of asymptomatic autoimmunity characterised by positivity for islet autoantibodies. Therefore, T helper cell responses that induce B cell activation are likely to play a critical role in the disease process. Here, we aimed to evaluate the role of a recently described subset, C-X-C motif chemokine receptor type 5-negative, programmed cell death protein 1-positive (CXCR5^−^PD-1^hi^) peripheral T helper (Tph) cells, in human type 1 diabetes.

**Methods:**

The phenotype of blood CXCR5^−^PD-1^hi^ CD4^+^ T cells was analysed by multicolour flow cytometry. The frequencies of circulating CXCR5^−^PD-1^hi^ T cells were analysed in a cohort of 44 children with newly diagnosed type 1 diabetes, 40 autoantibody-positive (AAb^+^) at-risk children and 84 autoantibody-negative healthy control children, and the findings were replicated in a separate cohort of 15 children with newly diagnosed type 1 diabetes and 15 healthy control children.

**Results:**

Circulating CXCR5^−^PD-1^hi^ Tph cells share several features associated with B cell helper function with circulating CXCR5^+^PD-1^hi^ follicular T helper (Tfh) cells. Moreover, the frequency of circulating Tph cells was increased in children with newly diagnosed type 1 diabetes, especially in those who are positive for multiple autoantibodies. Importantly, circulating Tph cells were also increased in autoantibody-positive at-risk children who later progressed to type 1 diabetes.

**Conclusions/interpretation:**

Our results demonstrate that circulating CXCR5^−^PD-1^hi^ Tph cells are associated with progression to clinical type 1 diabetes. Consequently, Tph cells could have potential both as a biomarker of disease progression and as a target for immunotherapy in type 1 diabetes.

**Electronic supplementary material:**

The online version of this article (10.1007/s00125-019-4936-8) contains peer-reviewed but unedited supplementary material, which is available to authorised users.

## Introduction

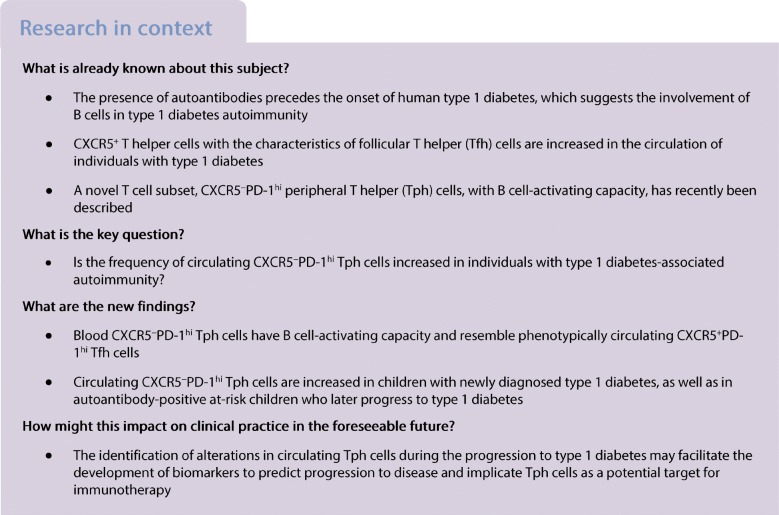



Type 1 diabetes is a T cell-mediated autoimmune disease characterised by beta cell destruction and dysfunction [1]. Autoantibodies produced by B cells are currently the best available biomarker for predicting human type 1 diabetes. Individuals positive for at least two islet autoantibodies have around 50% risk of developing type 1 diabetes within the next 5 years [2]. Despite the predictive potential of autoantibodies, it remains unclear whether autoreactive B cells have a direct pathological effect in the pathogenesis of type 1 diabetes. However, several studies have shown that B cells are abundant in the pancreatic islets of some individuals with type 1 diabetes, especially in those who are diagnosed at a young age and therefore likely have aggressive autoimmunity [3–5]. Moreover, one clinical trial has demonstrated a partial preservation of C-peptide levels after B cell depletion by rituximab [6].

Antibody production by B cells is strongly dependent on the help provided by helper T cells, especially CXCR5^+^ follicular T helper (Tfh) cells [7, 8]. We and others have demonstrated that circulating Tfh cells appear to be increased in individuals with type 1 diabetes [9–11]. Our study additionally suggested that this increase occurs close to the clinical diagnosis of the disease and only in individuals positive for multiple autoantibodies at diagnosis [11].

Recently, a novel population of CXCR5^−^PD-1^hi^ CD4^+^ T cells, coined peripheral T helper (Tph) cells, was shown to be strongly expanded both in the synovium and in the peripheral blood of individuals with seropositive rheumatoid arthritis [12]. These CXCR5^−^PD-1^hi^ Tph cells appear phenotypically similar to CXCR5^+^PD-1^hi^ Tfh cells since they express factors associated with B cell help, including IL-21 and inducible T cell costimulator (ICOS) and are capable of providing B cell help in vitro. However, instead of expressing CXCR5 that enables Tfh cells to home to lymphoid follicles, Tph cells express higher levels of chemokine receptors, such as C-C motif chemokine receptor 2 (CCR2), C-X3-C motif chemokine receptor 1 (CX3CR1) and CCR5, which direct migration to inflamed sites. Consequently, they are thought to play an important role in supporting B cell responses in inflamed tissues, complementing in this way the role of Tfh cells in lymphoid organs [13].

In the current study, we used samples from a large follow-up study of children to analyse whether, in addition to circulating Tfh cells, CXCR5^−^PD-1^hi^ Tph frequencies are altered during the development of type 1 diabetes.

## Methods

### Study participants

The study cohort has been described in detail previously [11]. In brief, samples analysed in this study were collected between October 2013 and January 2016. In total, we analysed 44 children with newly diagnosed type 1 diabetes (within a week of diagnosis, age 9.0 ± 3.6 years), 40 autoantibody-positive (AAb^+^) children (age 9.3 ± 4.7 years) and a control group of 84 autoantibody-negative healthy children of similar age (age 9.4 ± 3.8 years). The AAb^+^ children and healthy control children participated in the Finnish Type 1 Diabetes Prediction and Prevention (DIPP) follow-up study and had HLA types associated with increased risk for T1D. All the samples were paired (i.e. a blood sample from an age- and HLA-matched healthy control child was drawn, processed and analysed on the same day as the sample from an AAb^+^ child or child with type 1 diabetes). Of the AAb^+^ children, 15 were diagnosed with type 1 diabetes 7–37 months (mean ± SD 23 ± 10 months) after sampling (progressors) and 25 had not progressed to clinical disease (non-progressors) during the 3–5 years after sampling. An independent cohort of 15 children with newly diagnosed type 1 diabetes and 15 control children of similar age who were not part of the original cohort were recruited for the validation experiments. The study was approved by local ethics committees in the participating university hospitals (decisions 1.12.1994 and 375/13.02.00/2016). All children participating in the study and/or their legal guardians provided written informed consent, as mandated by the Declaration of Helsinki.

### Flow cytometric analyses

Immunostaining for surface and intracellular markers was performed as previously described [11] and the antibodies used are listed in ESM Table 1. In some experiments, T cell subsets were first isolated by flow cytometric sorting (FACSAria III, BD Biosciences, San Jose, CA, USA) and stimulated for 5 h with 50 ng/ml phorbol myristic acid (PMA; Sigma-Aldrich, St Louis, MO, USA), 1 μg/ml ionomycin (Sigma-Aldrich) and 3 μg/ml brefeldin A (Ebioscience, San Diego, CA, USA). The samples were acquired on FACSCanto II (BD Biosciences) or Cytoflex S (Beckman Coulter, Indianapolis, IN, USA) flow cytometers and the flow cytometry data was analysed using FlowJo software v10 (BD Biosciences). Coded samples were used throughout, and the flow cytometric analyses were performed blinded to the clinical classification of the sample.

### T cell and B cell co-cultures

The co-culture approach has been described in detail previously [11]. In brief, different T cell subsets as well as naive (CD20^+^IgD^+^CD27^−^) or memory (CD20^+^CD27^+^) B cells were flow-cytometrically sorted from peripheral blood mononuclear cells of healthy donors, and T cells and B cells were co-cultured together at a 1:10 ratio in the presence of 1 μg/ml *Staphylococcus* enterotoxin B (SEB) and 5 μg/ml lipopolysaccharide (LPS; both from Sigma-Aldrich) for 7 days before flow cytometric analyses (ESM Table 1 and ESM Fig. 1).

### Statistical analyses

Statistical analyses were performed using Prism software (GraphPad Software, San Diego, CA, USA). When comparing differences between groups either Mann–Whitney *U* test or Kruskal–Wallis test with Dunn’s multiple comparison test was used. Wilcoxon test was used when analysing paired samples. Relationships between different results were examined using Spearman correlation coefficient. *p* < 0.05 was considered to indicate statistical significance.

## Results

### Circulating CXCR5^−^PD-1^hi^ Tph cells express factors associated with B cell helper function and resemble circulating CXCR5^+^PD-1^hi^ Tfh cells

**Fig. 1:**
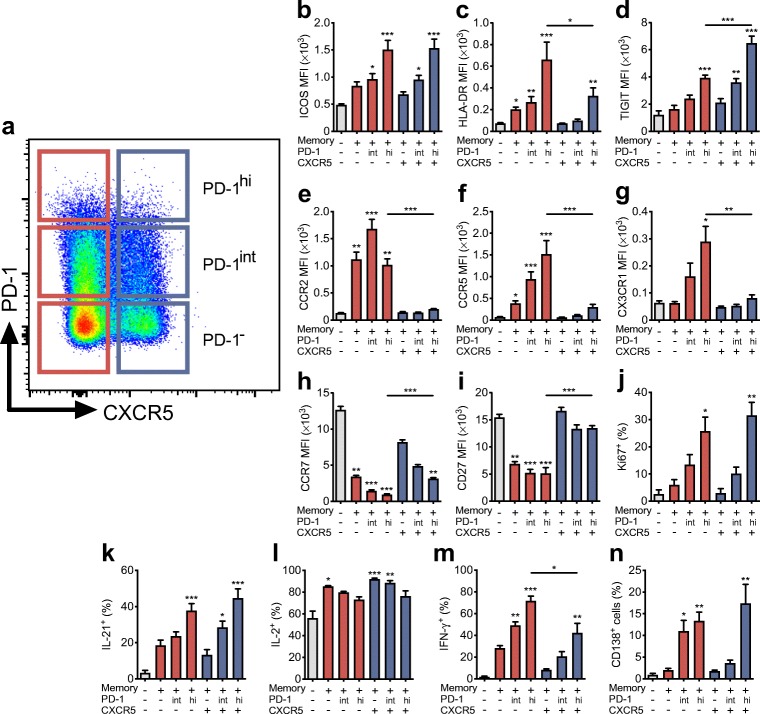
CXCR5^−^PD-1^hi^ Tph and CXCR5^+^PD-1^hi^ Tfh CD4^+^ memory T cell subsets share features associated with B cell helper function. (**a**) A representative flow cytometric staining of peripheral blood memory T cells (gated as CD3^+^CD4^+^CD45RA^−^) subdivided into CXCR5^−^ (red) and CXCR5^+^ (blue), and further into PD-1^−^, PD-1^int^ and PD-1^hi^ subsets. (**b**–**n**) The expression of different surface markers (**b**–**i**) and the proliferation marker Ki67 (**j**), the production of cytokines after PMA and ionomycin stimulation (**k**–**m**) and the capacity to induce memory B cell differentiation to plasma cells in a co-culture of the different subsets (**n**). The results are expressed as mean ± SEM geometric mean fluorescence intensity (MFI) values or percentage positive from four to eight experiments, each performed with cells from different healthy donors. For cytokine production and B cell co-culture experiments, the T cell subsets were flow-cytometrically sorted before the analyses. *p<0.05, **p<0.01 and ***p<0.001 compared with naive CD4^+^ T cells (grey bars), or CXCR5^–^PD-1^hi^ vs CXCR5^+^PD-1^hi^ subsets where indicated; Kruskal–Wallis test with Dunn’s post hoc test

Based on the expression of CXCR5 and programmed cell death protein 1 (PD-1), human peripheral blood memory CD4^+^ T cells can be subdivided into PD-1^−^, PD-1^int^ and PD-1^hi^ subsets that are either CXCR5^−^ or CXCR5^+^ (Fig. 1a). The PD-1^hi^ subsets are rare, typically comprising only 0.5–2% of total memory CD4^+^ T cells. First, we analysed the expression of markers that are associated with B cell helper function, differentiation status or homing capacity on these memory CD4^+^ T cell subsets. In line with previously published results [12], both the CXCR5^−^PD-1^hi^ and CXCR5^+^PD-1^hi^ subsets expressed high levels of ICOS, HLA-DR and T cell immunoreceptor with Ig and ITIM domains (TIGIT) (Fig. 1b–d), with higher expression levels of HLA-DR and lower expression of TIGIT observed in CXCR5^−^PD-1^hi^ compared with CXCR5^+^PD-1^hi^ (Fig. 1c,d). Moreover, these subsets differed in their expression of chemokine receptors and markers of differentiation, with CXCR5^−^PD-1^hi^ cells expressing higher levels of CCR2, CCR5 and CX3CR1 but lower levels of CCR7 and CD27 than CXCR5^+^PD-1^hi^ cells (Fig. 1e–i). Consequently, a larger fraction of CXCR5^−^PD-1^hi^ than CXCR5^+^PD-1^hi^ cells reside within the effector memory T cell compartment (ESM Fig. 1). Both CXCR5^−^PD-1^hi^ and CXCR5^+^PD-1^hi^ subsets expressed high levels of Ki67 ex vivo, suggesting that they contain recently activated cells proliferating in vivo (Fig. 1j). After PMA and ionomycin stimulation, the frequencies of CXCR5^−^PD-1^hi^ and CXCR5^+^PD-1^hi^ cells producing IL-21 and IL-2 were comparable, but clearly higher and lower, respectively, compared with the PD-1^−^ subsets (Fig. 1k,l). A greater proportion of CXCR5^−^PD-1^hi^ cells produced IFN-γ compared with CXCR5^+^PD-1^hi^ cells (Fig. 1m). Finally, we directly compared the capacity of the different T cell subsets to activate B cells in co-culture assays in vitro (ESM Fig. 1). Interestingly, both CXCR5^−^PD-1^hi^ and CXCR5^+^PD-1^hi^ cells were efficient in activating memory B cells into plasma cells, with CXCR5^−^PD-1^int^ cells also demonstrating this capacity (Fig. 1n and ESM Fig. 1). In turn, the CXCR5^+^PD-1^−^ and CXCR5^+^PD-1^int^ subsets were most efficient in activating naive B cells into plasmablasts (ESM Fig. 1). Of note, all memory T cell subsets were equally expanded during a 7 day co-culture with memory B cells, excluding the possibility that high PD-1 expression is associated with an exhausted phenotype (ESM Fig. 1). Collectively, we demonstrate here that circulating CXCR5^−^PD-1^hi^ Tph cells appear to share several characteristics with circulating CXCR5^+^PD-1^hi^ Tfh cells, with high expression of markers associated with B cell helper capacity, such as ICOS, TIGIT and IL-21, and comparable capacity to activate memory B cells in vitro.

### Circulating CXCR5^−^PD-1^hi^ Tph cells are increased in frequency in children with newly diagnosed type 1 diabetes and in autoantibody-positive children who later progressed to clinical disease

**Fig. 2:**
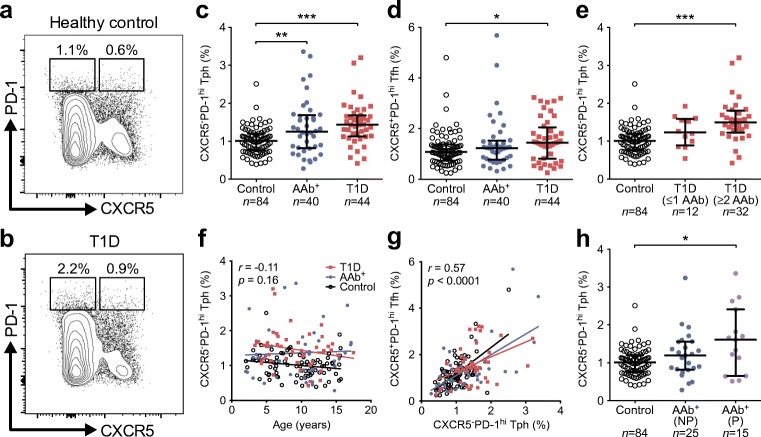
Higher frequency of circulating CXCR5^−^PD-1^hi^ Tph cells in children with newly diagnosed type 1 diabetes and in autoantibody-positive at-risk children who progressed to clinical disease. (**a**, **b**) Representative example of pairwise analyses of CXCR5^−^PD-1^hi^ Tph and CXCR5^+^PD-1^hi^ Tfh cells (as a percentage of total memory CD4^+^ T cells) from a healthy control child (**a**) and a child with type 1 diabetes (T1D, **b**). (**c**, **d**) The frequencies of CXCR5^−^PD-1^hi^ Tph (**c**) and CXCR5^+^PD-1^hi^ Tfh (**d**) cells in healthy control children, autoantibody-positive (AAb^+^) children and children with newly diagnosed type 1 diabetes. (**e**) CXCR5^−^PD-1^hi^ Tph cell frequencies in children with newly diagnosed type 1 diabetes stratified according to the number of biochemical autoantibodies (insulin autoantibodies [IAA], GAD antibodies [GADA] and islet antigen 2 antibodies [IA-2A]) at the time of sampling. (**f**, **g**) The frequency of CXCR5^−^PD-1^hi^ Tph cells within memory CD4^+^ T cells did not correlate with age (**f**) but did correlate with the frequency of CXCR5^+^PD-1^hi^ Tfh cells (**g**). Correlation was calculated by pooling all samples analysed and is expressed together with *p* values next to the individual plots. (**h**) The frequencies of CXCR5^−^PD-1^hi^ Tph cells in AAb^+^ children who did not progress (NP) or progressed (P) to type 1 diabetes. Median values with interquartile range are shown. *p<0.05, **p<0.01 and ***p<0.001; Kruskal–Wallis test with Dunn’s post hoc test

To analyse whether changes in circulating CXCR5^−^PD-1^hi^ Tph cell frequencies are altered in human type 1 diabetes, we re-analysed data from our large published study evaluating the frequencies of CXCR5^+^ Tfh cells at different stages of type 1 diabetes progression [11]. Since the gating of PD-1^hi^ cells in different samples is not clearly defined, to minimise analytical bias we only included paired samples from the original study where samples from AAb^+^ children or children with newly diagnosed type 1 diabetes were drawn, processed and analysed together with a paired sample from an autoantibody-negative child of similar age on the same day (Fig. 2a,b).

The frequency of CXCR5^−^PD-1^hi^ Tph cells was increased in both children with type 1 diabetes and in AAb^+^ children (Fig. 2c). In line with our published results [11], the frequency of CXCR5^+^PD-1^hi^ Tfh cells was, however, only increased in children with type 1 diabetes (Fig. 2d). These results were confirmed by a strict pairwise analysis of the samples processed and analysed in parallel on the same day (ESM Fig. 2). Of note, the frequencies of CXCR5^−^PD-1^int^ and CXCR5^+^PD-1^int^ T cells did not differ between the study groups (ESM Fig. 2), excluding the possibility that the increase in PD-1^hi^ Tph and Tfh subsets is caused by a general increase in PD-1 expression in children with type 1 diabetes or AAb^+^ children. Interestingly, the frequency of CXCR5^−^PD-1^hi^ Tph cells was only increased in children with type 1 diabetes who were positive for two or more autoantibodies (Fig. 2e), a phenomenon that was also observed in CXCR5^+^PD-1^hi^ Tfh cells (ESM Fig. 2 and [11]).

The frequency of CXCR5^−^PD-1^hi^ Tph cells within memory CD4^+^ T cells did not clearly change with the age of the children (Fig. 2f). In contrast, the frequency of CXCR5^+^PD-1^hi^ Tfh cells decreased with age (ESM Fig. 2 and [11]). Of note, the frequencies of CXCR5^−^PD-1^hi^ Tph and CXCR5^+^PD-1^hi^ Tfh cells correlated significantly (*r* = 0.57, *p* < 0.0001; Fig. 2g).

Importantly, we have follow-up data on the development of type 1 diabetes in the AAb^+^ children after the samples were analysed. When we divided the group of AAb^+^ children into progressors and non-progressors to type 1 diabetes, we were interested to observe that the frequency of CXCR5^−^PD-1^hi^ Tph cells was only increased in the children that later progressed to type 1 diabetes (Fig. 2h and ESM Fig. 2). Other factors potentially influencing the risk of disease progression, such as age, HLA class II genotype and autoantibody status, were comparable between the two groups (ESM Table 2).

In conclusion, we demonstrate that both CXCR5^−^PD-1^hi^ Tph and CXCR5^+^PD-1^hi^ Tfh cells are increased in the blood of children with newly diagnosed type 1 diabetes, especially in children with multiple autoantibodies. However, only Tph cells and not Tfh cells appear to be increased in AAb^+^ children who later progressed to clinical disease.

### TIGIT expression is elevated in CXCR5^−^PD-1^hi^ Tph cells from children with newly diagnosed type 1 diabetes

**Fig. 3:**
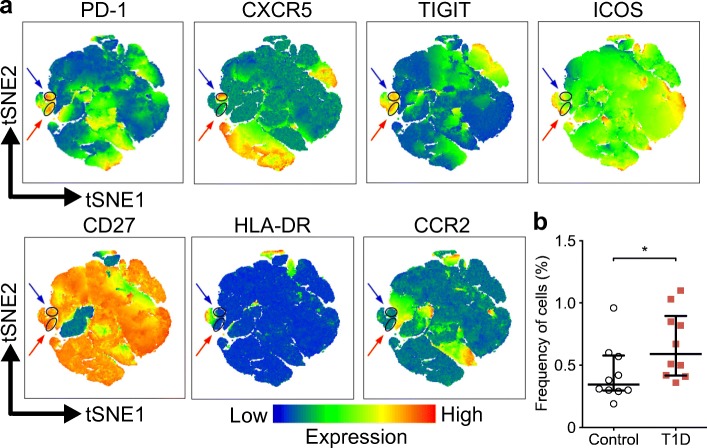
CXCR5^−^PD-1^hi^TIGIT^+^ T cells are increased in children with newly diagnosed type 1 diabetes. (**a**) tSNE plots of memory CD4^+^ T cells (pre-gated on viable CD3^+^CD4^+^CD45RA^−^ cells) from a combined batch of 10 children with type 1 diabetes (T1D) and 10 healthy children as the control group. Colour indicates surface expression levels of labelled markers. The manually gated CXCR5^−^PD-1^hi^ cluster (red arrows) also expresses high levels of TIGIT and co-localises with the CXCR5^+^PD-1^hi^ cluster (blue arrows). (**b**) The frequency of memory CD4^+^ T cells within the CXCR5^−^PD-1^hi^ cluster in 10 children with type 1 diabetes compared with 10 healthy children (control group) analysed in batch. Median values with interquartile range are shown. *p<0.05; Mann–Whitney *U* test

To validate our results, we performed an extended flow cytometric analysis on an independent cohort of 15 children with newly diagnosed type 1 diabetes and 15 children of similar age as a healthy control group. For this analysis, we added additional markers previously suggested to be differentially expressed by Tph cells (TIGIT, ICOS, HLA-DR, CCR2 and CD27) [12] to determine whether a certain combination of these phenotypic markers would better define the CXCR5^−^PD-1^hi^ cell population expanded in children with type 1 diabetes. In line with published data [12], two-dimensional visualisation of memory CD4^+^ T cells by t-distributed stochastic neighbour embedding (tSNE) clustered the main populations of CXCR5^−^PD-1^hi^ and CXCR5^+^PD-1^hi^ cells in close proximity, suggesting a similar multidimensional phenotype (Fig. 3a). Both the CXCR5^−^PD-1^hi^ and CXCR5^+^PD-1^hi^ clusters appeared to contain cells expressing high levels of TIGIT. Importantly, the frequency of cells within the CXCR5^−^PD-1^hi^ cluster was higher in children with type 1 diabetes than in healthy controls (Fig. 3b). We confirmed this finding through manual gating, comparing the expression levels of the different phenotypic markers on CXCR5^−^PD-1^hi^ cells (ESM Fig. 2). The only marker that was differentially expressed by CXCR5^−^PD-1^hi^ cells from children with vs without type 1 diabetes was TIGIT (ESM Fig. 2). Consequently, an increased frequency of CXCR5^−^PD-1^hi^TIGIT^+^ circulating Tph cells was observed in children with type 1 diabetes (ESM Fig. 2). In conclusion, the extended analysis of the validation cohort confirms that CXCR5^−^PD-1^hi^ Tph cells are increased in the blood of children with type 1 diabetes and suggests that TIGIT may be a useful auxiliary marker to define circulating Tph cells.

## Discussion

In the current study, we demonstrate that circulating CXCR5^−^PD-1^hi^ memory CD4^+^ T cells display a B cell helper phenotype ex vivo and appear to be expanded in children with newly diagnosed type 1 diabetes as well as in autoantibody-positive children who later progressed to clinical disease.

Expansion of CXCR5^−^PD-1^hi^ T cells both in the synovium and peripheral blood was first described in individuals with rheumatoid arthritis [12]. To our knowledge, our study is the first to describe the expansion of these cells in peripheral blood of individuals with type 1 diabetes. In the previous study, CXCR5^−^PD-1^hi^ T cells were coined peripheral T helper (Tph) cells in order to differentiate them from the better-established subset of CXCR5^+^PD-1^hi^ follicular T helper (Tfh) cells [12]. Due to the capacity of Tph cells to activate B cells and recruit them to the tissues through the production of the C-X-C motif chemokine ligand 13 (CXCL13), they are hypothesised to play an important role in supporting B cell responses and the formation of ectopic lymphoid structures in tissues under inflammatory conditions, complementing in this way the role of Tfh cells in lymphoid organs [13]. A CXCR5^−^PD-1^hi^ population highly similar to Tph cells has also been identified within tumour-infiltrating lymphocytes in individuals with breast cancer [14]. Importantly, a recent paper employing HLA class II tetramers to directly characterise gluten-specific T cells in the blood and gut of individuals with coeliac disease demonstrated that the pathogenic antigen-specific T cells in coeliac disease also have a CXCR5^−^PD-1^hi^ phenotype with high expression levels of IL-21 and CXCL13 transcripts, highly reminiscent of Tph cells [15]. In the same paper, CXCR5^−^PD-1^hi^ T cells were also shown to be expanded in the blood of individuals with systemic sclerosis and systemic lupus erythematosus, further suggesting that the expansion of Tph cells in blood is a feature shared by several autoimmune diseases [15].

Based on both our current and previously published data [12], circulating CXCR5^−^PD-1^hi^ Tph cells are clearly a population with heterogeneous marker expression. Understanding this heterogeneity better and identifying additional markers to more unambiguously define circulating Tph cells associated with autoimmunity is a major research goal for the future. Our initial analyses indicate that TIGIT, an immunomodulatory receptor also expressed at high levels by CXCR5^+^PD1^hi^ Tfh cells in blood and tonsils (Fig. 1; [12, 16]), shows promise as a candidate auxiliary marker for the identification of potentially pathogenic Tph cells in individuals with type 1 diabetes. It is also unclear whether circulating Tph cells, or Tfh cells, in individuals with type 1 diabetes-associated autoimmunity contain T cells recognising beta cell antigens or whether they represent expansions of ‘bystander’ T cells. Although technically challenging, future studies employing HLA class II tetramers may shed light on this question, as recently demonstrated in coeliac disease [15].

The developmental relationship between Tfh cells and Tph cells is currently unclear: does a subset of Tfh cells differentiate into Tph cells during the germinal centre reaction or do Tph cells derive from separate peripheral effector T cells that acquire B cell helper function [13]? The strong correlation between circulating Tph and Tfh cell frequencies observed in our study would support the hypothesis for a common developmental pathway.

An obvious caveat of our study is that we could only analyse Tph cells in blood samples. The frequency of CXCR5^−^PD-1^hi^ T cells in peripheral blood is low, on average around 1% of memory CD4^+^ T cells, and the increase in circulating Tph cell frequencies in children with type 1 diabetes and AAb^+^ children and healthy control children is modest at best. It is important to note, however, that the frequencies of circulating Tph cells in the peripheral blood of individuals with rheumatoid arthritis are also similarly low, even though they constitute on average more than 25% of all CD4^+^ T cells in synovial fluid or synovial tissue [12]. Therefore, it is possible that also in type 1 diabetes Tph cells could constitute a major subpopulation of CD4^+^ T cells at the level of inflamed islets.

In addition to the well-characterised predictive potential of autoantibodies in type 1 diabetes, several lines of evidence support the importance of B cell autoimmunity also directly at the level of inflamed islets. In the NOD mouse model, B cell infiltration and the generation of ectopic lymphoid structures are a general feature of autoimmune insulitis [17–20]. Moreover, CXCL13, a chemokine produced by Tph cells [12, 14], appears to be expressed at high levels in the islets [20, 21]. Finally, CXCR5^−^ICOS^+^ IL-21-producing T cells that bear high resemblance to Tph cells have been reported to infiltrate the islets in NOD mice [22]. Although ectopic lymphoid structures are absent in human islets, a B cell infiltration is observed also in inflamed human islets, especially in individuals that are very young at onset of type 1 diabetes and thus likely have a more aggressive disease course [3–5]. An important goal for future studies is to address whether CD4^+^ T cells in inflamed human islets that harbour B cells display a phenotype characteristic of Tph cells.

In conclusion, we demonstrate here that CXCR5^−^PD-1^hi^ Tph cells are expanded in the circulation before and at the diagnosis of type 1 diabetes. Together with previous reports on Tfh cells [9–11], our current results provide further support for a critical role for interactions between T cells and B cells in the pathogenesis of type 1 diabetes and provide a rationale that targeting these interactions could be therapeutically effective. Moreover, since circulating Tph cells appear to be more predominantly expanded than Tfh cells in autoantibody-positive children, they also show potential for further evaluation as a biomarker of disease progression and for monitoring the effects of immunotherapy.

## Electronic supplementary material

ESM (PDF 685 kb)

## Data Availability

The datasets generated and/or analysed during the study are available from the corresponding author on reasonable request.

## Abbreviations

AAb^+^: Autoantibody-positive
CCR: C-C motif chemokine receptor
CX3CR: C-X3-C motif chemokine receptor
CXCL: C-X-C motif chemokine ligand
CXCR: C-X-C motif chemokine receptor
ICOS: Inducible T cell costimulator
PD-1: Programmed cell death protein 1
PMA: Phorbol myristic acid
TIGIT: T cell immunoreceptor with Ig and ITIM domains
Tfh: Follicular T helper
Tph: Peripheral T helper
tSNE: t-Distributed stochastic neighbour embedding
