# Supplementary material for: Circulating CXCR5−PD-1hi peripheral T helper cells are associated with progression to type 1 diabetes
Source: Diabetologia. 2019 Jul 3;62(9):1681–8. doi: 10.1007/s00125-019-4936-8 (PMC6677711; doi:10.1007/s00125-019-4936-8)
Supplement: Supplementary file 1 — (PDF 685 kb) [file 125_2019_4936_MOESM1_ESM.pdf]

ESM Table 1 Fluorescent monoclonal antibodies used in the study

**Antigen**	**Clone**	**Fluorochrome**	**Manufacturer**
CD3	SK7	APC-F750	Biolegend, San Diego, CA
CD4	RPA-T4	APC-H7	BD Biosciences
		APC-F750	Biolegend
		FITC	Biolegend
		PE-Cy7	Biolegend
		BV510	Biolegend
CD45RA	H100	FITC	Biolegend
		PerCP-Cy5.5	Biolegend
CCR7	G043H7	A647	Biolegend
CD27	M-T271	BV421	Biolegend
		APC-R700	BD Biosciences
		PerCP-Cy5.5	Biolegend
CD25	BC96	PE-Cy7	Biolegend
PD-1	EH12.2H7	PE	Biolegend
ICOS	C398.4A	A647	Biolegend
		APC	Biolegend
HLA-DR	L243	BV421	Biolegend
CXCR3	G025H7	A647	Biolegend
CXCR5	J252D4	PerCP-Cy5.5	Biolegend
	RF8B2	BB515	BD Biosciences
CX3CR1	2A9-1	A647	Biolegend
CCR2	K036C2	BV421	Biolegend
		BV605	Biolegend
CCR5	J418F1	BV421	Biolegend
IFN-γ	4S.B3	APC	Biolegend
IL-2	MQ1-17H12	PE-Cy7	Biolegend
IL-21	3A3-N2	BV421	BD Biosciences
CD19	HIB19	BV510	Biolegend
CD20	2H7	APC	BD Biosciences
IgD	IA6-2	A488	Biolegend
CD38	HIT2	BV421	Biolegend
CD138	MI15	PE	Biolegend
CD14	M5E2	BV510	Biolegend
CD56	HCD56	BV510	Biolegend
Zombie Aqua	Live/Dead		Biolegend

Each antibody was titrated before use to ensure an optimal signal-to-noise ratio.

ESM Table 2 Characteristics of autoantibody-positive at-risk children

	**Nonprogressors (n=25)**	**Progressors (n=15)**
**Age at sampling (years +/-SD)**	9.3 +/- 4.8	9.4 +/- 4.6
**Age at first AAb (years +/-SD)**	4.8 +/- 4.0	3.8 +/- 2.8
**HLA class II genotype[1] (%)**		
DQ2.5/x	2/25 (8.0%)	4/15 (26.7%)
DQ8/x	18/25 (72.0%)	6/15 (40.0%)
DQ2.5/DQ8	5/25 (20.0%)	5/15 (33.3%)
**Number of autoantibodies[2] (%)**		
1	9/25 (36.0%)	4/15 (26.7%)
2	13/25 (52.0%)	8/15 (53.3%)
3	3/25 (12.0%)	3/15 (20.0%)

[1]DQ2.5 = DQA1*05-DQB1*02, DQ8 = DRB1*04-DQA1*03-DQB1*0302, x ≠ DQB1*0602
[2] number of biochemical autoantibodies (IAA, GADA and IA-2A) detected at sampling

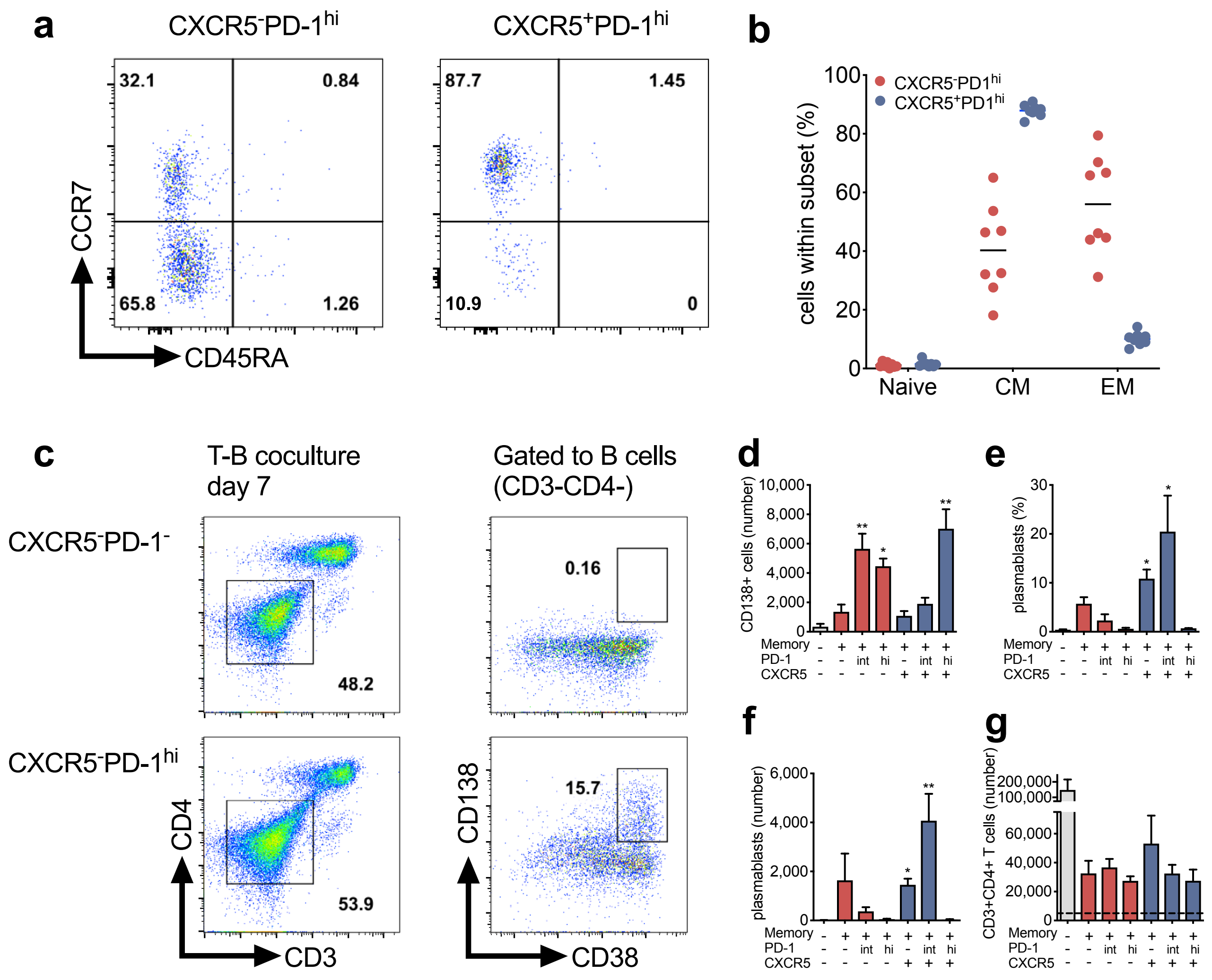

ESM Figure 1 (**a**) Representative staining of CD45RA and CCR7 on CD3$^+$CD4$^+$ CXCR5$^-$PD-1^{hi} and CXCR5$^+$PD-1^{hi} T cells. (**b**) The distribution of CXCR5$^-$PD-1^{hi} and CXCR5$^+$PD-1^{hi} T cells within naïve (CD45RA$^+$CCR7$^+$), central memory (CM, CD45RA$^-$CCR7$^+$) and effector memory (EM, CD45RA$^-$CCR7$^-$) subsets in eight healthy donors analyzed. For the T-cell and B-cell coculture assays different sorted T-cell subsets were cocultured with either memory (CD27$^+$) or naive (CD27$^-$) B cells and the frequencies of T cells (CD3$^+$CD4$^+$), plasma cells (CD3$^-$CD4$^-$CD38^{hi}CD138$^+$) and plasmablasts (CD3$^-$CD4$^-$CD20^{low}CD38^{hi}) were analyzed after 7 days. (**c**) A representative example of the assay with CXCR5$^-$PD-1$^-$ (top panels) and CXCR5$^-$PD-1^{hi} memory CD4+ T cells (bottom panels) cocultured with memory B cells is shown. (**d**) Absolute numbers of CD138$^+$ plasma cells in cocultures with memory B cells were determined with counting beads. (**e, f**) Frequencies and absolute numbers of plasmablasts in cocultures with naive B cells, and (**g**) the absolute number of T cells in cocultures with memory B cells. The dashed line indicates the input number of T cells in the coculture (5000 T cells). The results are expressed as mean +/-SEM of four separate experiments performed with cells from different healthy donors. *p<0.05 and ** p<0.01 compared to naive CD4+ T cells (grey bars); Kruskal–Wallis test with Dunn's post hoc test

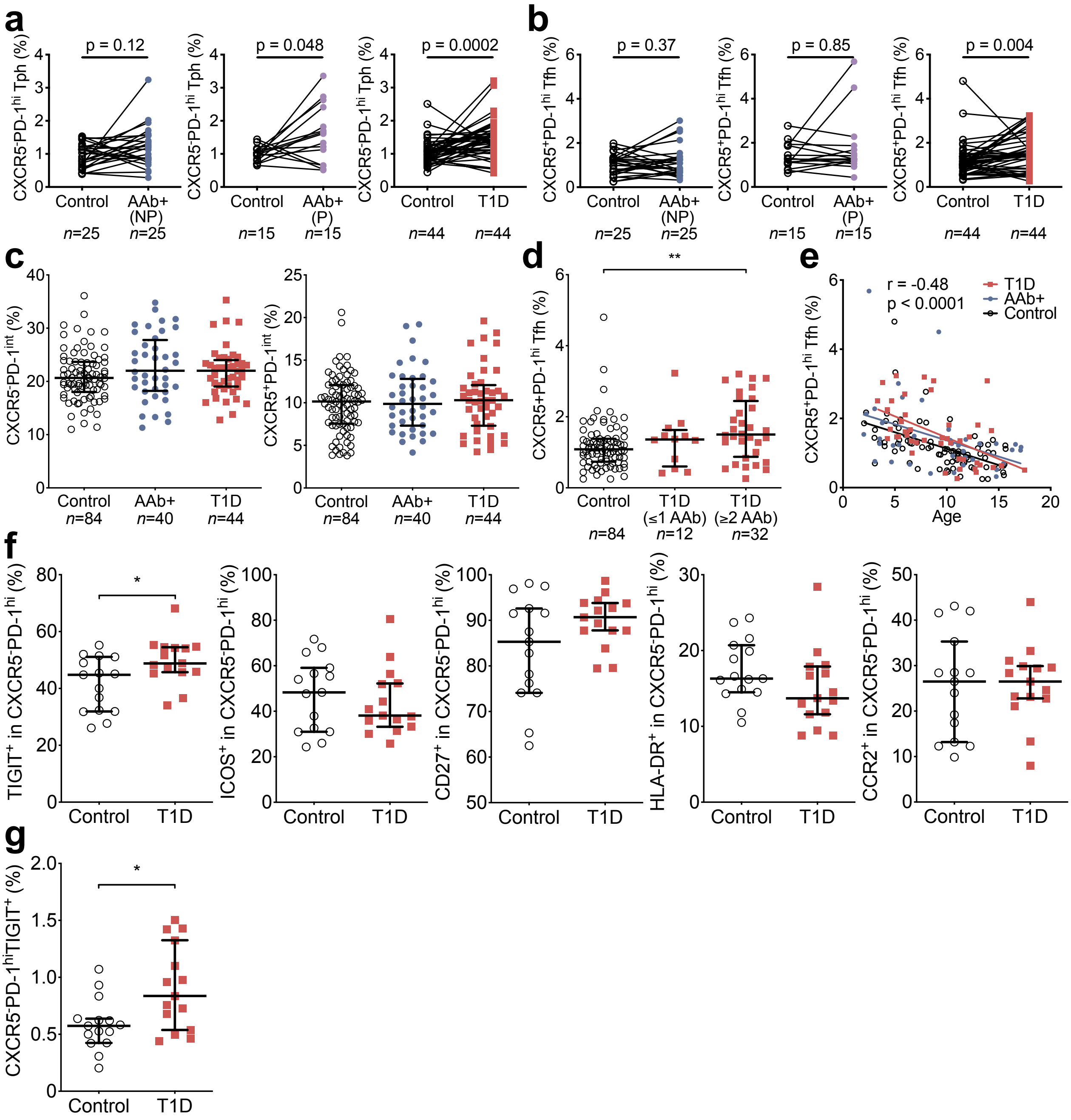

ESM Figure 2 (**a**,**b**) Pairwise analyses of CXCR5$^-$PD-1^{hi} Tph and CXCR5$^+$PD-1^{hi} Tfh cell frequencies. Samples from autoantibody-positive at-risk children (AAb+) who did not progress (NP) or progressed (P) to type 1 diabetes (T1D) and from children with newly diagnosed T1D were compared to samples from age-matched healthy children (control) processed and analyzed in parallel. *p* values from paired Wilcoxon tests are indicated. (**c**) The frequencies of CXCR5$^-$PD-1^{int} and CXCR5$^+$PD-1^{int} memory CD4+ T cells in the study groups. (**d**) CXCR5$^+$PD-1^{hi} Tfh cell frequencies in children with newly diagnosed T1D stratified based on the number of biochemical autoantibodies (IAA, GADA and IA-2A) detected in their blood at the time of sampling. (**e**) The frequency of CXCR5$^+$PD-1^{hi} Tfh cells correlates negatively with age. Correlation was calculated by pooling all samples analyzed and is expressed together with the *p* value next to plot. (**f**) Expression of surface markers (TIGIT, ICOS, CD27, HLA-DR and CCR2) on manually gated CXCR5$^-$PD-1^{hi} T cells in a total of 15 children with T1D and 15 healthy controls (validation cohort). (**g**) Manually gated CXCR5$^-$PD-1^{hi}TIGIT$^+$ memory T cell frequencies in 15 children with T1D and 15 healthy controls. Median values with interquartile range are shown. *$p<0.05$ and **$p<0.01$; Kruskal–Wallis test with Dunn's post hoc test or Mann–Whitney *U* test
